# Evaluation of cosmetic efficacy of lychee seed fermentation liquid

**DOI:** 10.1186/s40643-024-00818-9

**Published:** 2024-10-31

**Authors:** Yaqian Yan, Hao Fu, Yuling Tang, Tiantian Huang, Xun Zou, Ning Su, Dongdong Wang, Changtao Wang, Meng Li

**Affiliations:** 1 Beijing Key Laboratory of Plant Resource Research and Development, College of Light Industry Science and Engineering, Beijing Technology and Business University, Beijing, People’s Republic of China; 2 Institute of Cosmetic Regulatory Science, Beijing Technology and Business University, Beijing, People’s Republic of China; 3 Guilin Sanjin Pharmaceutical Co.,Ltd, Beijing, People’s Republic of China; 4 Chinese Academy of Inspection and Quarantine, Beijing, People’s Republic of China

**Keywords:** Lychee seed, Fermentation, Antioxidant, Anti-inflammatory, Security

## Abstract

**Background:**

Lychee seeds were fermented by three kinds of bacteria (*Lactobacillus plantarum, Saccharomyces cerevillus* and *ganoderma lucidum mycelium*), and two effective strains were selected by two indexes of activity content and antioxidant, so as to further verify whether lychee seeds have waste multiplication effect and can protect cells damaged by oxidation from anti-inflammatory, anti-aging and safety.

**Results:**

The contents of polyphenols, flavonoids and proteins in the solution fermented by *Ganoderma lucidum mycelium* did not increase, thus affecting the antioxidant capacity of the solution was far less than that of the water extract. The active content of the other two fermentation solutions was higher than that of the water extract, and the ability of scavenging free radicals of the two solutions increased with the increase of the volume fraction. At the cellular level, the two fermentation solutions showed repair effects on UVA-induced damaged cells. The contents of type I collagen (COL-1), total antioxidant capacity and ELN were increased, the contents of reactive oxygen species and MDA were decreased, and the expressions of inflammatory factors IL-6, TNF-a, iNOS and COX-2 were decreased in HaCaT cells. From the gene level, the mRNA contents of IL-6, TNF-a, Caspase-3, Caspase-9, Bax and Bcl were significantly decreased. The test of chick embryo chorioallantoic membrane (HTET CAM) showed that there was no bleeding and litchi seed fermentation liquid was not irritating.

**Conclusions:**

Therefore, two kinds of litchi seed fermentation can be used as natural plant raw materials for cosmetics, and have strong antioxidant, anti-inflammatory and anti-aging functions on skin, and also have good human safety.

**Graphical Abstract:**

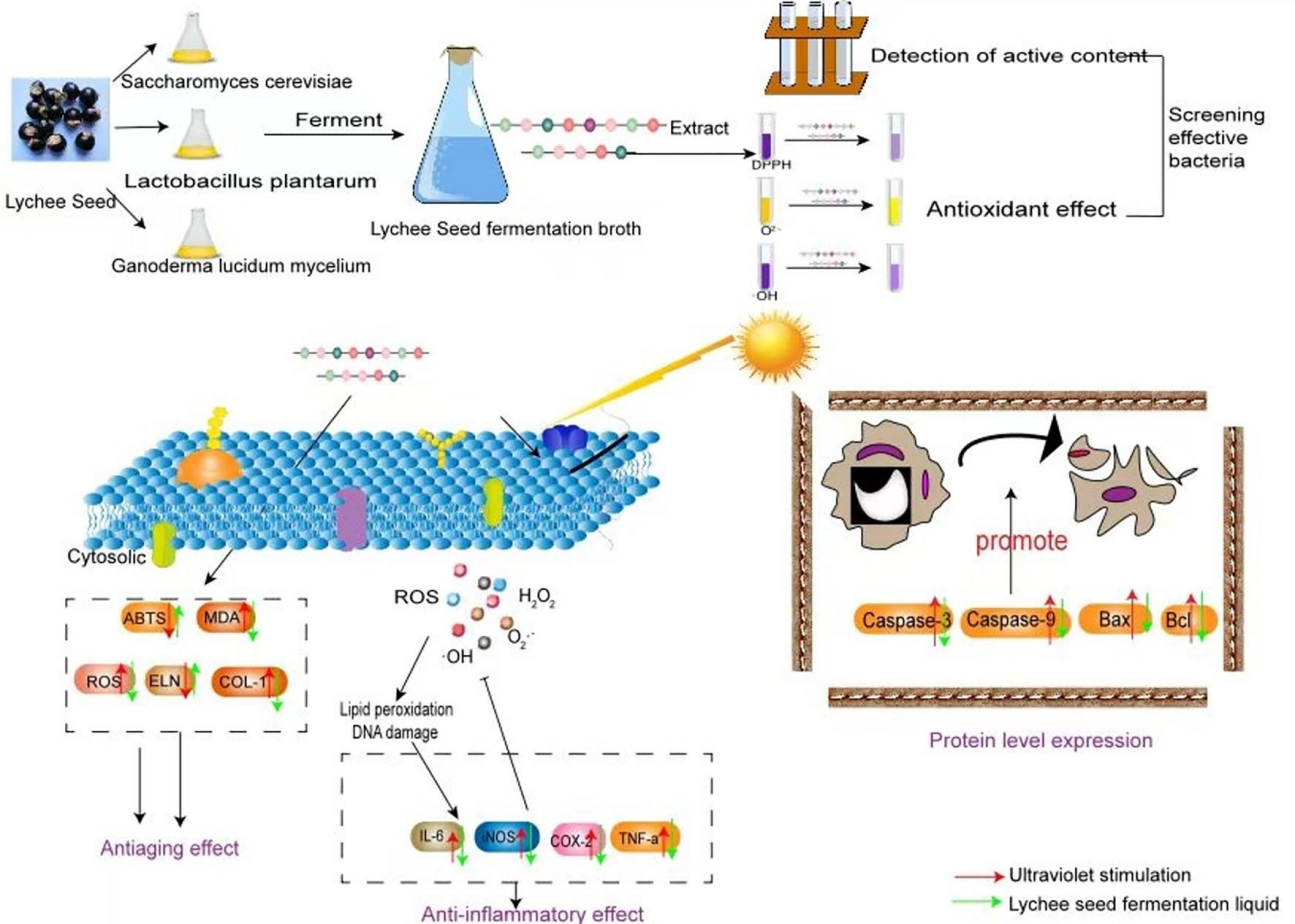

## Introduction

Lychee seed is a constituent of the lychee plant in the Sapindaceae family. Remove the pericarp and fleshy aril of lychee, wash and dry in the sun to obtain lychee seed (De Oliveira et al.^2024^). Lychee seeds will release waste water containing impurities in the landfill process, which will penetrate into surface water or groundwater and cause black odor, release a large amount of greenhouse gases and cause environmental pollution (Huang et al. 2024). Lychee seed is rich in starch and nutrients, which can be used as medicinal materials. As a traditional Chinese medicinal material, Lychee seed is warm, sweet and slightly bitter, it has the effects of qi clearing, cold removing and pain relieving, etc. (Ma et al. 2020 and Wu et al. 2020). Lychee seed exhibits an pivotal role in the development of health care products and pharmaceutical research, however its research in the field of cosmetics is not extensive, so this article mainly introduces the role of lychee seed fermentation liquid in anti-inflammatory, antioxidant and safety. The data shows: Compared with rice, protein peptide powder, persimmon leaf, tortoise flower and other fermentation raw materials, lychee seed raw materials are easy to obtain and cheap, only 0.016 yuan /g.

Because fermentation can transform microorganisms into new substances under specific conditions, and the operation is simple and non-dangerous, fermentation technology is widely used in various fields, but it has not been widely used in cosmetics (Yang et al. 2018). A large number of experiments have proved that the activity of the raw material after fermentation is increased, and the anti-aging, anti-inflammatory and anti-oxidation effects are enhanced. For example, compared with water extract, peony fermentation liquid has better antioxidant capacity and less irritability in terms of its active content and free radical scavenging ability (Zhang et al. 2022a, b). Salvia miltiorrhizae fermentation liquid has obvious protective effect on the model after hydrogen peroxide damage, which can enhance catalase activity, reduce melanin synthesis, and achieve whitening effect (Mo et al. 2021). After fermentation, the flavonoid content of Lycium barbarum branches is significantly increased, the content of reactive oxygen species in cells is significantly removed, and IL-8 inflammatory factors are inhibited, thus playing a potential anti-inflammatory role (Zhang et al. 2022a, b). In this experiment, Lychee seeds fermented by ganoderma lucidum mycelium did not effectively improve the level of active substances and efficacy, so they did not conform to the fermentation concept. Skin plays a protective, sensory and immune function for the body (Chen et al. 2023). Skin problems arise when stimulated by external factors that cause oxidative stress, promote the release of inflammatory factors and damage the skin barrier (Chen et al. 2022). The skin barrier is the body’s “umbrella”, damage to it can lead to disease (DeVore et al. 2020; Chen et al. 2019). To avoid diseases, it’s essential to find relevant raw materials to solve skin problems.

The efficacy of lychee seed extract has been reported, but the effect of lychee seed fermentation broth obtained from *Lactobacillus plantarum, saccharomyces cerevisiae* and *Ganoderma lucidum mycelium* on cell models has not been reported. Therefore, three fermentation broths were the target of this project. By measuring the content of bioactive substances, the level of free radical scavenging, and the content of inflammatory factors released in the raw materials, the results highlight the important role of lychee seeds as a raw material for the development of microbial fermentation products.

## Experiments

### Reagents and instruments

Reagents: HaCaT cells and fibroblast, purchased from Shaoyuan (Shanghai) Biotechnology Co., LTD, lychee seed, purchased in Guangxi Province, China; concentrated sulfuric acid, phenol, bovine serum protein, folinol reagent, copper sulfate, anhydrous ethanol, sodium nitrate, NaCl (Sinophosphoric Chemical Reagent Co., Ltd.); DPPH (Sigma Corporation); *S. cerevisiae* (Courtesy of the Food Brewing Institute); *L. plantarum* (Shandong Zhongke Jiayi Biological Engineering Co.); Total Antioxidant Capacity Detection Kit (ABTS), ROS Detection Kit, Intracellular Catalase Detection Kit, Malondialdehyde Detection Kit, Type I Collagen Detection Kit, Human Elastin ELISA Kit, TNF-α, COX-2, iNOS, IL-6 ELISA kits (Sangong Bioengineering (Shanghai) Co., Ltd.); DMEM medium, fetal bovine serum, fibroblast growth additive, penicillin/streptomycin, 0.05% (including EDTA) trypsin (Gibco); Na2HPO4, KH2PO4, citric acid (Beijing Chemical Plant); NaCl (Sinopharm Chemical Reagent Co., Ltd.); KCl (Tianjin Guangfu Technology Development Co., Ltd.); MRS AGAR medium (Guangzhou Bailing Biotechnology Co., Ltd.).

### Method

#### Cell culture

HaCaT cells and fibroblasts were placed in high-sugar DMEM medium in a 37 ℃ incubator (Rayward), and the cell density reached more than 80% for passage or experiment.

#### Cell modeling

HaCaT cells were cultured according to 2.2.1, inoculated in a 6-well culture plate with 2 × 10^5^ cells per well,stimulated with 2 mL lipopolysaccharide (300 µg/mL), and cultured with lychee seed fermentation broth with 5% volume fraction for 24 h, then the superserum was collected, store in − 80 for future use.

The cultured cells in 2.2.1 were inoculated into 96-well plates with 10,000 cells per well. When the number of cells reached more than 90%, UVA (7 J/cm^2^) was irradiated, the samples were cleaned twice with PBS, and the supernuant was collected after 24 h.

#### Preparation of lychee seed water extract and fermentation broths

30 g of lychee seeds were weighed, added to 3L of deionized water, sterilized at 121 °C for 30 min and added to a fermenter, then 100 mL of *S. cerevisiae* liquid, 100 mL of *L. plantarum* liquid and 100 mL of *Ganoderma lucidum mycelium* fermentation liquid were added respectively. (The lychee seed water extract was free of bacteria).

The samples were cultured at 37 °C for 24 h and sterilized, After centrifugation (4800 r/min 20 min), the supernatant was taken and applied to the experiment. The obtained solutions were lychee core water extract (L0), lychee core fermentation (L1) prepared by *S. cerevisiae* fermentation, lychee core fermentation (L2) prepared by *L. plantarum* fermentation and lychee core fermentation (L3) prepared by *Ganoderma lucidum mycelium* fermentation.

#### Determination of lychee seed fermentation broth content

Protein content: The experimental method was referred to (Loptien et al. 2024). 1 mL sample and 1 mL alkaline ketone solution were mixed for 10 min, then 4 mL folinol test solution was added to determine the light absorption value. (Shanghai One Hang Seng Science and Technology Co., Ltd.).

Polysaccharide content: Referring to (Wang et al. 2024a, b, c), Refer to Table 1 to add substances and complete the experiment.

**Table 1:** Reaction system

Sample	5% phenol solution	Concentrated sulfuric acid
1 mL	1 mL	5 mL
Incubate at 30°for 10 min		
OD490		

Flavonoid content: Refer to Dawuti et al. (2024), take 1 mL sample and add 0.3 mL 5% sodium nitrite, place it for 6 min, add 0.3 mL 10% aluminum nitrate, place it for 6 min, add 4 mL 1 mol/L NaOH and 0.4 mL water, shake it well, place it for 10 min, and measure absorbance at 510 mm.

Polyphenol content: References (Wang et al. 2024a, b, c) Experiments were carried out with reference to Table 2 reaction system.

**Table 2:** Reaction system

Sample	Distilled water	Folin-phenol test solution	26.7% Na_2_CO_3_ solution	Water
1 mL	1 mL	0.5 mL	1.5 mL	6 mL
Incubate at 37° for 2 h				
OD760				

#### Analysis of antioxidant efficacy of lychee seed fermentation broth in vitro

##### DPPH radical scavenging

DPPH experiment references (Yamauchi et al. 2024), the reaction system is shown in Table 3.

**Table 3:** Reaction system

L0	L1	L2	L3	无水乙醇
0.5 mL	0.5 mL	0.5 mL	0.5 mL	0.5 mL
DPPH (0.5 mL, concentration 0.0002%)				
Reaction for 30 min, OD517				

The calculation formula is as follow (1).

1 $${\text{DPPH }}\;{\text{free}}\;{\text{ radical}}\;{\text{ scavenging}}\;{\text{ rate }}\left( \% \right) = \left\{ {1 - \left( {T - \, T_{0} } \right)/\left( {C - C_{0} } \right)} \right\} \times 100\%$$

T—sample tube light absorption value; T_0_—sample background light absorption value; C-dpph tube absorption value; C_0_—solvent background absorption value.

##### Hydroxyl radical scavenging

0.5 mL 0.75 mol/L anhydrous ethanol solution of o-diazophane was poured into the test tube, and PBS, distilled water, ferrous sulfate and hydrogen peroxide solution were added (Wu et al. 2024)_,_ their light absorption values were measured at 536 nm. The hydroxyl clearance rate was calculated according to Formula (2).

2 $$Clearance \, rate\left( \% \right) = \left( {A_{sample} - A_{damage} } \right)/\left( {A_{no \, damge} - A_{damage} } \right) \times 100\%$$

A: Light absorption value.

##### Superoxide anion removal experiment

With reference to the experimental method in reference (Jing et al. 2021), Tris–HCl buffer of p H = 8.0, samples of different concentrations and a certain volume of anhydrous ethanol were successively added into a 5 mL centrifuge tube. After evenly mixing, 25μL and 30 mmol/L of pyrocatol solution were added, and after full mixing, OD values were measured every half minute at 325 nm. The superoxide anion clearance rate of the sample was calculated according to formula (3).

3 $$Clearance \, rate \, \left( \% \right) \, = \, \left( {V_{control} - \, V_{sample} } \right)/V_{control} \times \, 100\%$$

V_control_: control group pyrogallol autoxidation rate (△OD/min), V_sample_: sample group pyrogallol autoxidation rate (△OD/min).

#### Analysis of antioxidant effects in vivo

##### Fibroblast toxicity test (MTT)

100 μL MTT (1 mg/mL) solution was added to the 96-well plate containing fibroblasts, and after culture for 4 h, 150 μL DMSO was added to the discard solution, and the absorbents of each hole were measured at 490 nm after slow shaking for 10 min (Song et al. 2024 and Stindlova et al.^2024^). The cell survival rate was calculated according to Formula (4).

4 $$Cell \, viability \, = \, \left( {A_{determation} - \, A_{blank} } \right)/\left( {A_{control} - \, A_{blank} } \right)$$

A: Light absorption value.

##### Determination of oxidation index

Defrost the sample in 2.2.2 quickly. Total antioxidant capacity (TEAC), malondialdehyde (MDA) content, elastin (ELN) content, COL-I content, and Reactive oxygen species (ROS) content were measured by oxidation indexes which were operated according to the kit instructions.

#### Analysis of anti-inflammatory effects of lychee seed fermentation broth

##### Measurement of inflammatory factors

The sample in 2.2.2 was added to the 96-well plate containing the substrate, and the enzyme binding, color developing agent, and termination solution were added successively according to the TNF-α, IL-6, COX-2 and iNOS kit.

##### Determination of expression level of inflammatory factor genes

After HaCaT cells were treated with the samples, extract RNA, For the specific operation, refer to the instructions. Reverse transcription was performed according to the kit instructions. According to the gene sequence published by the National Center for Biotechnology Information of the United States, Primer Express software was used to design the specific primers for target genes.

#### Analysis of safety efficacy of lychee seed fermentation broth

##### Erythrocyte hemolysis experiment

For the specific experimental steps, refer to reference (Wang et al. 2024a, b, c). The sample solution of each concentration gradient was mixed with the red blood cell suspension at a ratio of 3:1, incubated for 60 min, centrifuged (10,000 × g) for 1 min, Measure the OD540 of the supernatant. The standard curve was drawn and the hemolysis rate was calculated according to Formula (5)。 According to denaturation ratio (L/D) = H50/D1, sample irritation can be judged.

5 $$Hemolysis \, ratio \, = \, \left( {A_{Sample} - \, A_{Negative} } \right)/A_{Complete \, hemolysis} - \, A_{Negative} ) \, \times \, 100\%$$

##### HET-CAM irritation test

The sample was directly penetrated into the chorionic membrane to observe changes in its toxic parameters (bleeding, hemagglutination, vasolysis, etc.). The end point score (ES) was calculated according to the end point evaluation method, and ES value represents sample irritation

### Data analysis

The experimental data were statistically analyzed by Excel, GraphPad Prism 8 for plotting, and IBM SPSS statistics 22 for significance T-test. Each experiment was conducted three times.

## Results

### Sample content

**Fig. 1:**
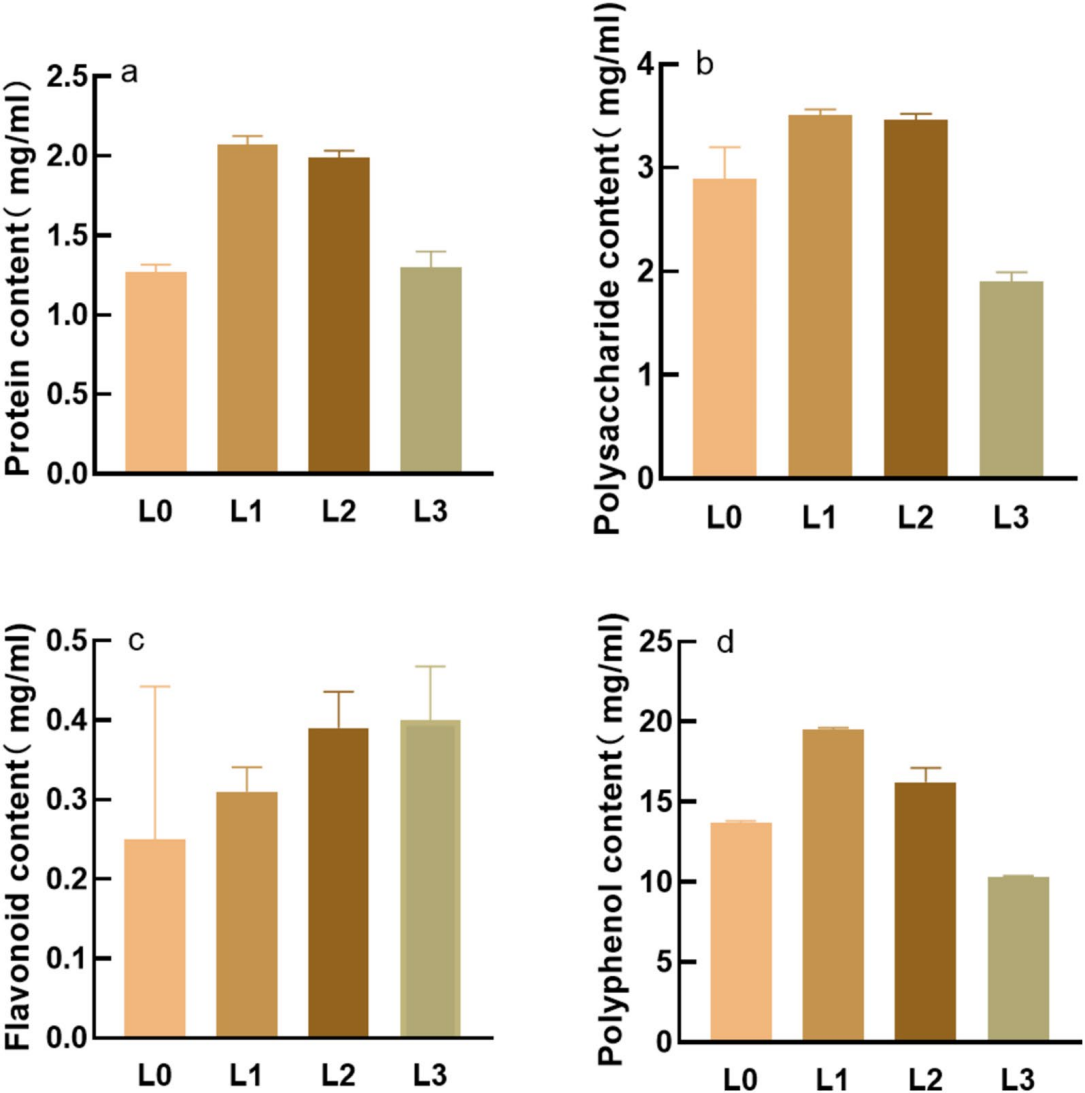
Sample activity content. **a** Protein content **b** Polysaccharide content **c** Flavonoid content d:Polyphenol content

There is a proportional relationship between active content and efficacy. Literature shows (Li et al. 2024)that after fermentation samples were analyzed by UPLC-Q/TOF–MS technology, flavonoids were the most, mainly flavonoids, flavonosides, flavonols and so on. The protein and polysaccharide contents of the sample are shown in Fig. 1a and b. Among the four samples, the protein and polysaccharide contents of L1 and L2 were higher (> 2.0 mg/mL), and much higher than that of L0, indicating that fermentation can activate the active content of the sample, but the active content of L3 was not higher than that of L0, which may be due to the inactivation of ganoderma lucidum mycelium, fermentation instability and other reasons. The flavonoid content of the sample is shown in Fig. 1c. The flavonoid content of 3 fermentation fluids was higher than that of L0, and L3 content was the highest (0.4 mg/mL), which was because the ganoderma lucidum mycelium contained flavonoid substances. The polyphenol content of the sample is shown in Fig. 1d. The content of L1 and L2 polyphenols is higher than that of L0, and polyphenols help to enhance the antioxidant effect.

### Antioxidant experiment results

**Fig. 2:**
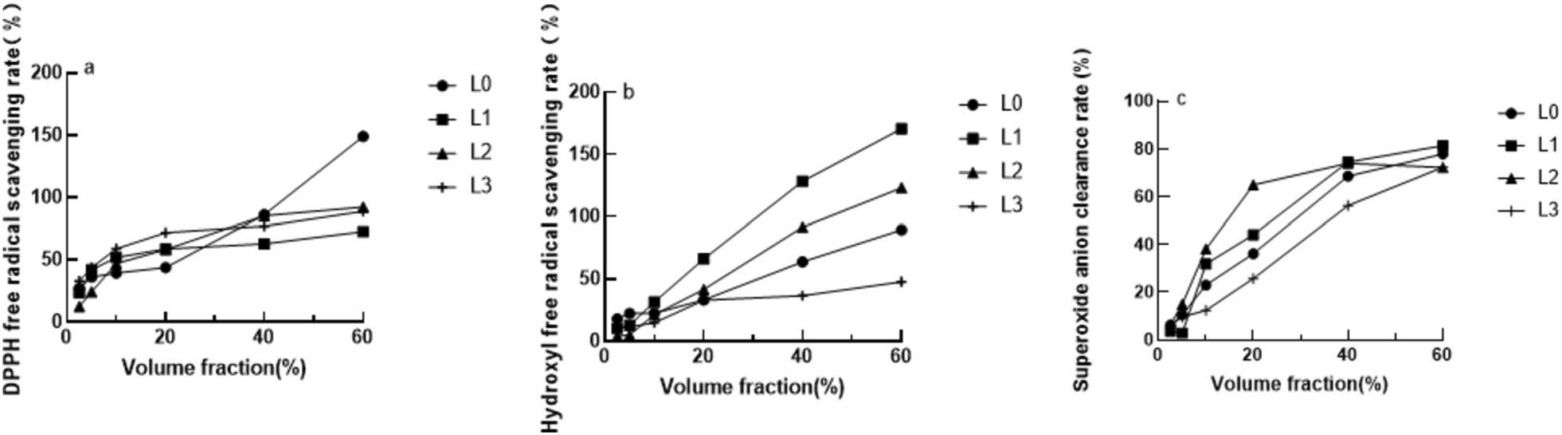
Sample clearance rates. **a** DPPH free radical scavenging experiment **b** Hydroxyl radical scavenging experiment **c** Superoxide anion radical scavenging experiment

The antioxidant effect in vitro is shown in Fig. 2. The scavenging effect of the sample on DPPH free radicals is shown in Fig. 2a. After the volume fraction of 20%, L0 showed an exponential growth, while the growth of the other three samples was slow and close.

The scavenging effect of the samples on hydroxyl free radicals is shown in Fig. 2b. The scavenging rate of the four samples increases exponentially with the increase of the volume fraction, and the scavenging rate of L1 and L2 is much higher than that of L0 and L3.

The scavenging rate of superoxide anion in the sample is shown in Fig. 2c, where the volume fraction reaches about 40%, and the clearance rate changes gently or decreases. The IC50 of L0, L1, L2, and L3 are 25.087, 21.617, 16.775, and 34.263, respectively. (IC50: that is, the semi-inhibitory concentration, the sample concentration required to achieve half the free radical scavenging effect, the smaller the IC50, the stronger the effect). L3 has the highest IC50 and the worst clearance effect, which may be related to its low activity content. Therefore, only L0, L1 and L2 were studied in subsequent experiments.

### Cell survival rate

**Fig. 3:**
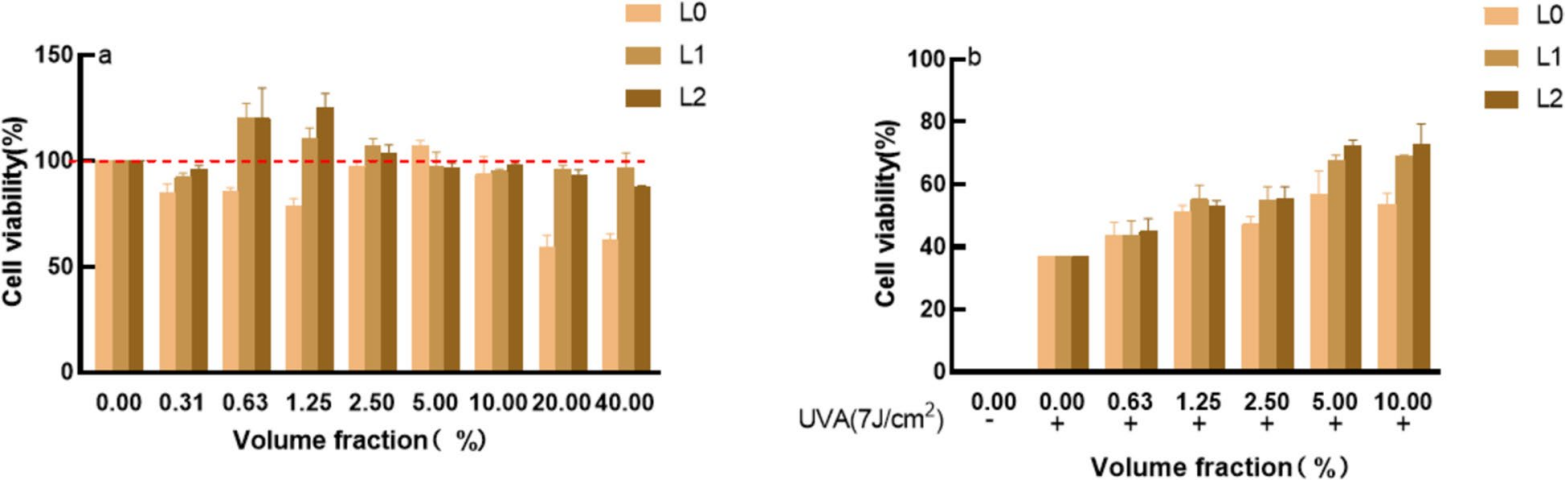
Effects of samples on cell activity. **a** the effects of the samples on cell activity **b** the protective effects of the samples on UVA-induced cell damage

Figure 3a shows the effects of the samples on cell activity. In the blank group without any samples, it was found that L0 had the highest toxicity with an IC_80_ equal to 4.492%. (IC80: The volume fraction of the sample at 80% cell survival). Fig. 3b shows the protective effects of the samples on UVA-induced cell damage. It can be seen that all samples had a protective effect. When the sample volume fraction was 5%, the cell survival rate was high, so the sample volume fraction was set at 5% for the follow-up experiments.

### Analysis of antioxidant efficacy of samples

#### Results of MTT toxicity test

**Fig. 4:**
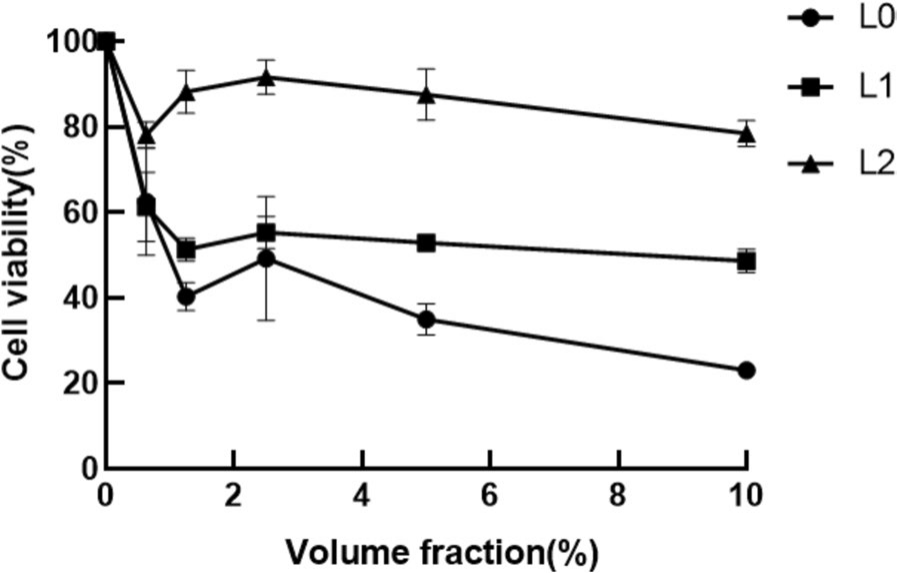
Effects of samples on cell activity

The effects of different samples on fibroblast activity are shown in Fig. 4. It can be seen from the curves in the figure that the samples had toxic effects on cells, and the higher the volume fraction, the stronger the toxicity. The IC80 of L0, L1 and L2 were 0.088%, 0.009% and 0.003%, respectively. It can be seen that the cytotoxic effect of samples L1 and L2 was less than that of L0, and it can be seen that fermentation can reduce the cytotoxic effect.

#### TEAC, MDA, ELN and ROS content of samples

The model group was the cell group irradiated only by UVA without adding samples, while the blank group was the cell group irradiated without adding samples.

**Fig. 5:**
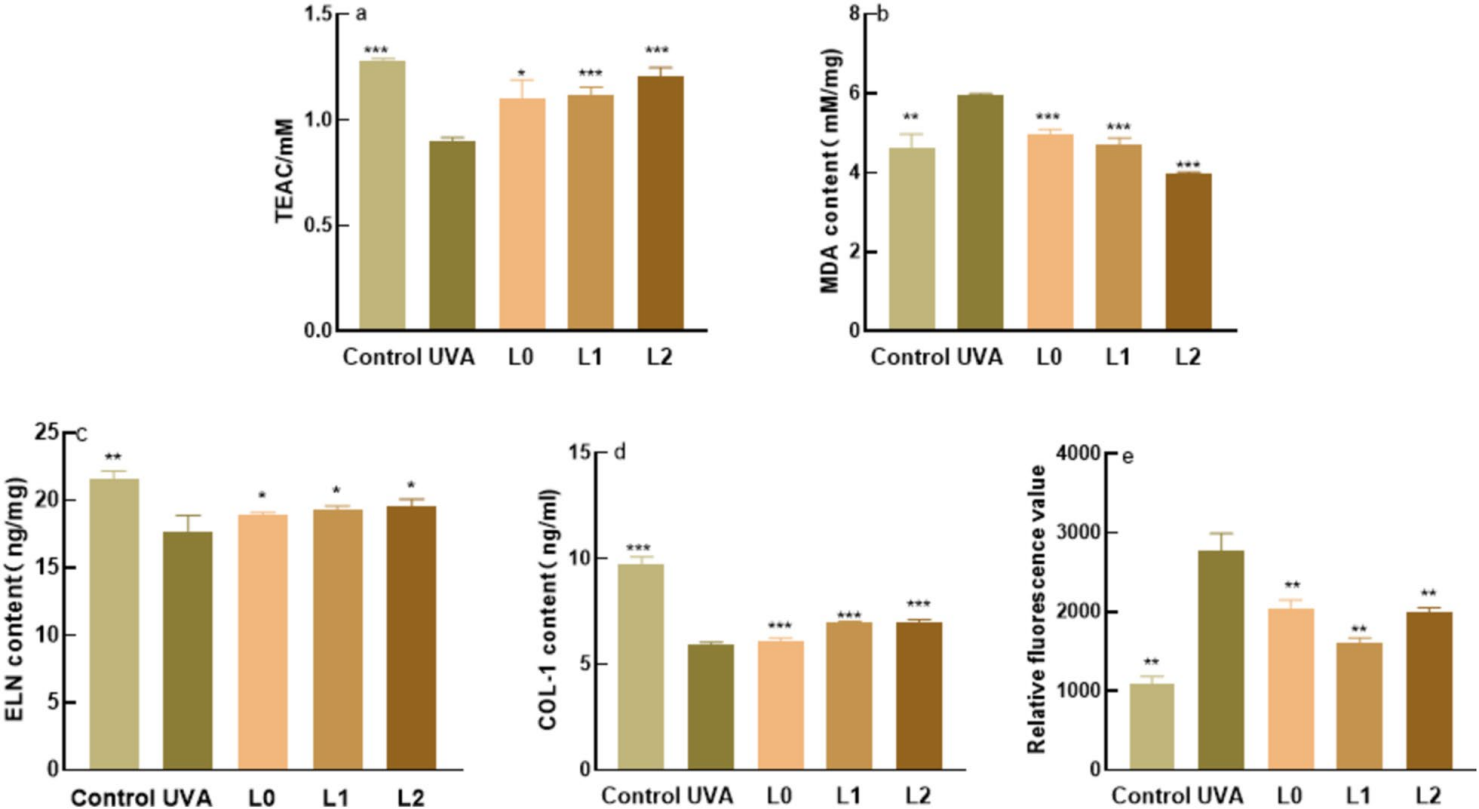
Contents of TEAC, MDA, ELN, COL-I and ROS in samples. **a** Total antioxidant capacity **b** MDA content **c** ELN content **d** COL-1 content **e** ROS content. *: P < 0.05 vs c (model group), **: P < 0.01 vs c (model group), ***: P < 0.001 vs c (model group). P < 0.05 is a statistically significant difference, P < 0.01 is an extremely statistically significant difference and P < 0.001 is a particularly statistically significant difference

TEAC content is shown in Fig. 5a, UVA irradiation reduces the total antioxidant capacity of the cells, the total antioxidant capacity of the three samples was reversed when the samples were applied to the cells, and the fermentation broth was more effective.

Malondialdehyde content (MDA) is shown in Fig. 5b, after UVA irradiation, cell peroxidation is serious, when the samples acted on the cells, L0, L1, L2 have extremely significant reduction of MDA content, reduce oxidation phenomenon.

Elastin content (ELN) and Type I collagen content (COL-1) are shown in Fig. 5c and d, ELN and COL-1 are important proteins in the skin, and their content is related to whether the skin is aging. UVA irradiation destroys the dermis and causes protein loss, when the samples acted on fibroblasts, ELN and COL-1 content were significantly increased.

Reactive oxygen species (ROS) content is an important signal to detect whether cells are damaged. The reactive oxygen species content is shown in Fig. 5e, after UVA irradiation reactive oxygen species content was increased dramatically, the sample group and the model group did statistical difference comparison found that the comparison, L0, L1, L2 had a significant reduction of reactive oxygen species content.

### Analysis of anti-inflammatory efficacy of samples

#### Analysis of expression level of inflammatory factor proteins

**Fig. 6:**
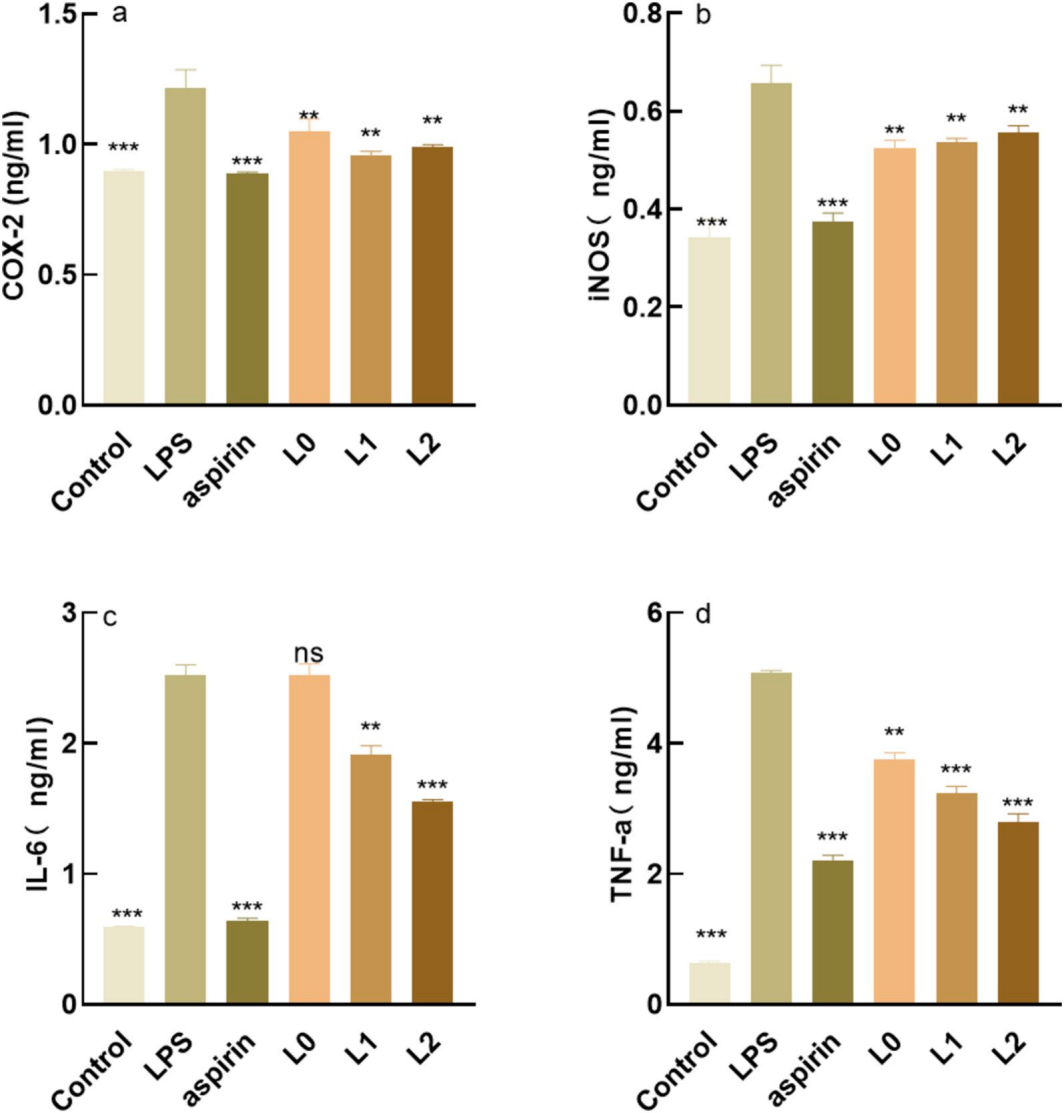
Expression of COX-2, iNOS, IL-6, TNF-a. **a** Cyclooxygenase-2 content (COX-2) **b** Inducible Nitric Oxide Synthase content (iNOS). **c** interleukin-6 content (IL-6) **d** tumor necrosis factor α content (TNF- α). *: P < 0.05 vs c (model group), **: P < 0.01 vs c (model group), ***: P < 0.001 vs c (model group), P < 0.05 is a statistically significant difference, P < 0.01 is an extremely statistically significant difference and P < 0.001 is a particularly statistically significant difference

The blank group was a HaCaT cell group without adding samples, the negative control group was an LPS-induced inflammatory injury group, and the positive control group was an aspirin cell group after inflammatory injury. The effects of the samples on inflammatory cytokines were detected. As shown in Fig. 6a, compared with the model group, L0 particularly significantly reduced the COX-2 content, while L2 significantly reduced the COX-2 content. As shown in Fig. 6b–d, all three samples significantly reduced the iNOS content, IL-6 content and TNF-a content.

#### Analysis of inflammatory factor gene expression level

**Fig. 7:**
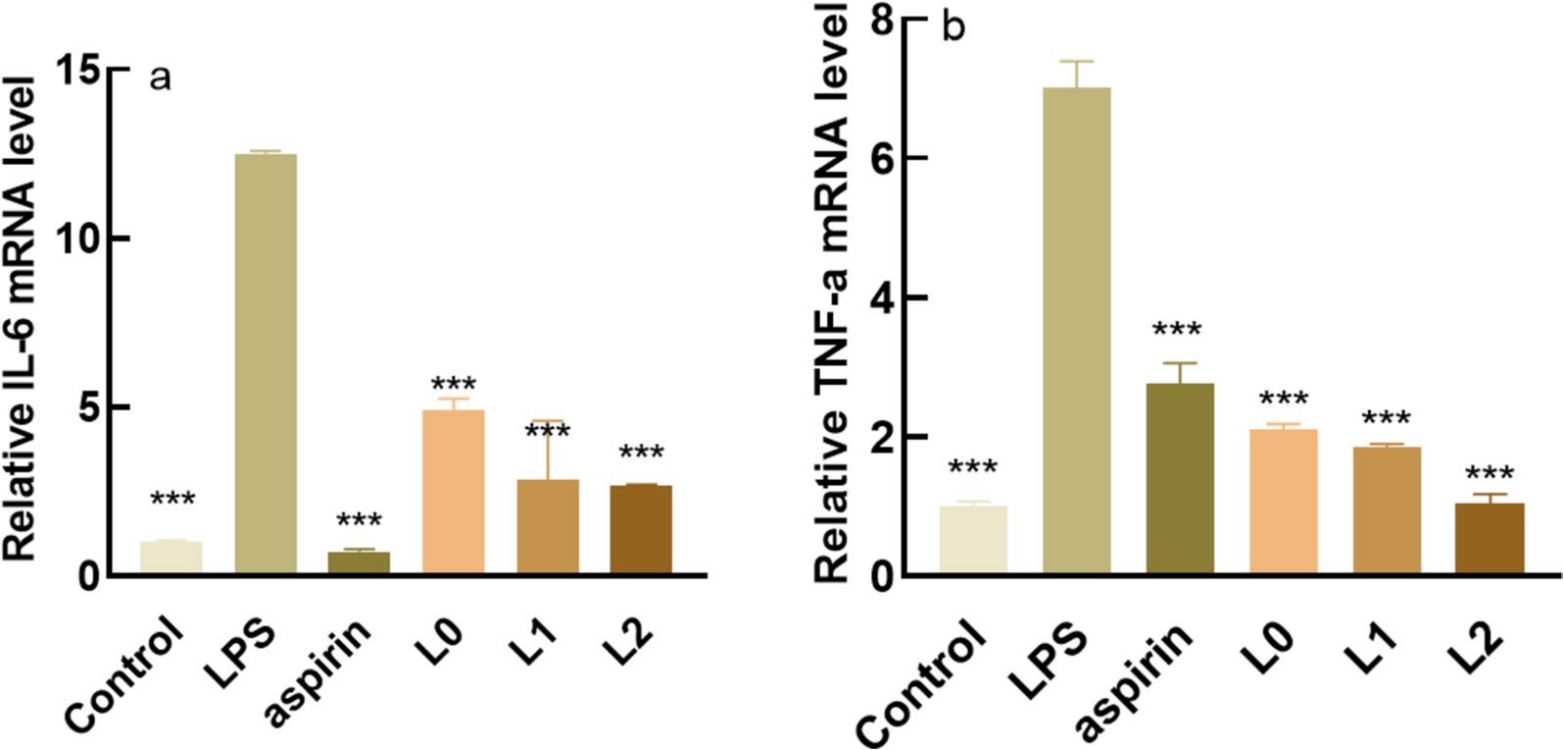
Expression of IL-6 and TNF-a gene. **a** Relative IL-6 mRNA level **b** Relative TNF-a mRNA level. *: P < 0.05 vs c (model group), **: P < 0.01 vs c (model group), ***: P < 0.001 vs c (model group), P < 0.05 is a statistically significant difference, P < 0.01 is an extremely statistically significant difference and P < 0.001 is a particularly statistically significant difference

Figure 7 shows the effects of the samples on the mRNA expression of inflammatory factors. As can be seen in Fig. 7a and b, L0, L1, and L2 significantly reduced the mRNA expression of IL-6 and TNF-a, with the effect of L2 being stronger than those of L0 and L1.

#### Expression of apoptotic molecules

**Fig. 8:**
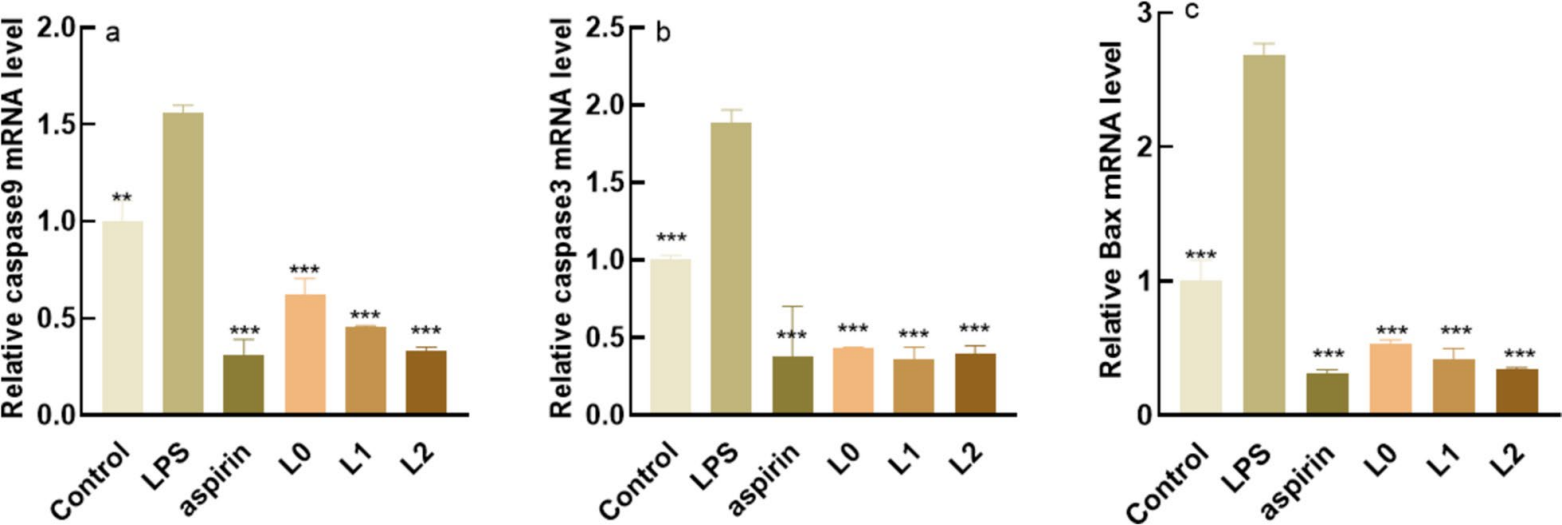
Expression of caspase-3, caspase-9, Bax. **a** Relative caspase3 mRNA level **b** Relative caspase9 mRNA level. **c** Relative Bax mRNA level. *: P < 0.05 vs c (model group), **: P < 0.01 vs c (model group), ***: P < 0.001 vs c (model group), P < 0.05 is a statistically significant difference, P < 0.01 is an extremely statistically significant difference and P < 0.001 is a particularly statistically significant difference

The Caspase family and Bax proteins are related to each other during the regulation process. Caspase-3, caspase-9 and Bax are apoptosis-promoting proteins, with caspase-3 being the major apoptosis-executing protease during apoptosis (Zhang et al. 2019). Fig. 8a and b show that L0, L1 and L2 had extremely significant effects on reducing the mRNA levels of Caspase-9 and Caspase-3, with similar values. L0, L1, and L2 can significantly reduce Bax mRNA level, with L2 having a better effect than L1 in Fig. 8c.

### Sample safety evaluation

#### Red blood cell hemolysis experiment

**Fig. 9:**
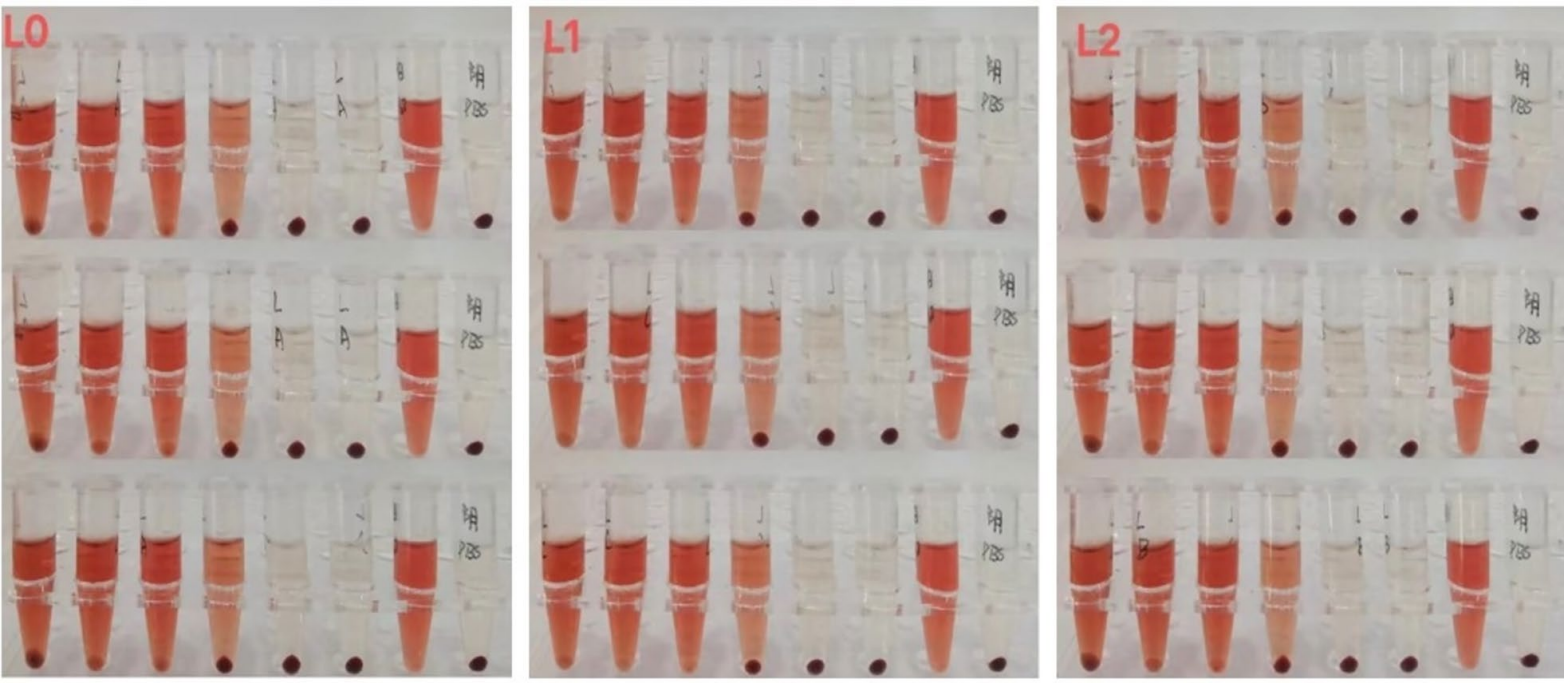
Red blood cell hemolysis test. Each group of samples were made in three parallel groups, from left to right, and the test tubes were: 100% sample, 80% sample, 60% sample, 40% sample, 20% sample, 10% sample, water, PBS. Positive control: water; Negative control: PBS

**Fig. 10:**
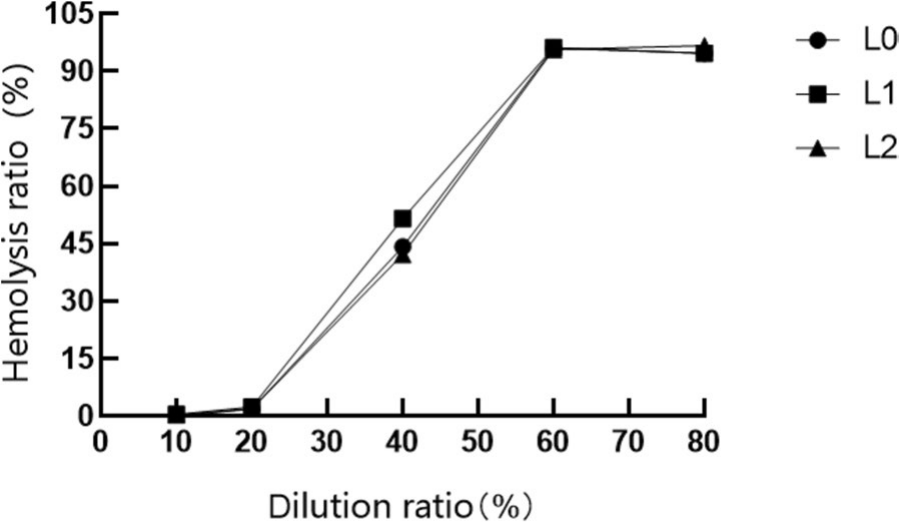
Sample hemolysis curve

The basic principle of red blood cell hemolysis test is to evaluate the damage of chemicals to eye tissue cells by measuring the amount of hemoglobin leakage and the degree of protein denaturation. As can be seen from Fig. 9, with the gradual decrease of sample concentration, the hemolysis rate becomes lower and lower, and the sample stimulation becomes smaller and smaller. This can be seen in Fig. 10, the hemolysis rate increases with the increase of sample dilution, and there is little difference between the hemolysis rate curves of the three samples.

#### Irritant evaluation

A stimulation evaluation table was made on the basis of the hemolysis curves of the samples, and the irritant evaluation of the stock solution is shown in Table 4 in terms of the H_50_, D_1_, and L/D values of each sample. (H_50_: Hemolysis rate. D_1_: Single denaturability, that is, the critical concentration of red blood cell discoloration under the action of sample hemolysis. L/D: cleavage/denaturation ratio, the ratio to determine the irritability of the sample.)

**Table 4:** Stimulation evaluation table

Number of samples	H_50_ (%)	D_1_ (%)	L/D	Stimulation evaluation
L0	53	39.89	1.33	Moderate irritation
L1	51.7	137.10	0.38	Irritation
L2	46.9	129.47	0.36	Irritation

The red blood cell test (RBC) is used to evaluate the irritation of chemicals on eye tissues (Wang et al. 2024a, b, c) The experimental results showed that all three samples had certain irritability, but there was water in the fermentation broth stock solution which meant that the experimental results would be affected to a certain extent, so the chicken embryo chorioallantoic membrane test (HET-CAM) was added.

#### HET-CAM irritation score

**Fig. 11:**
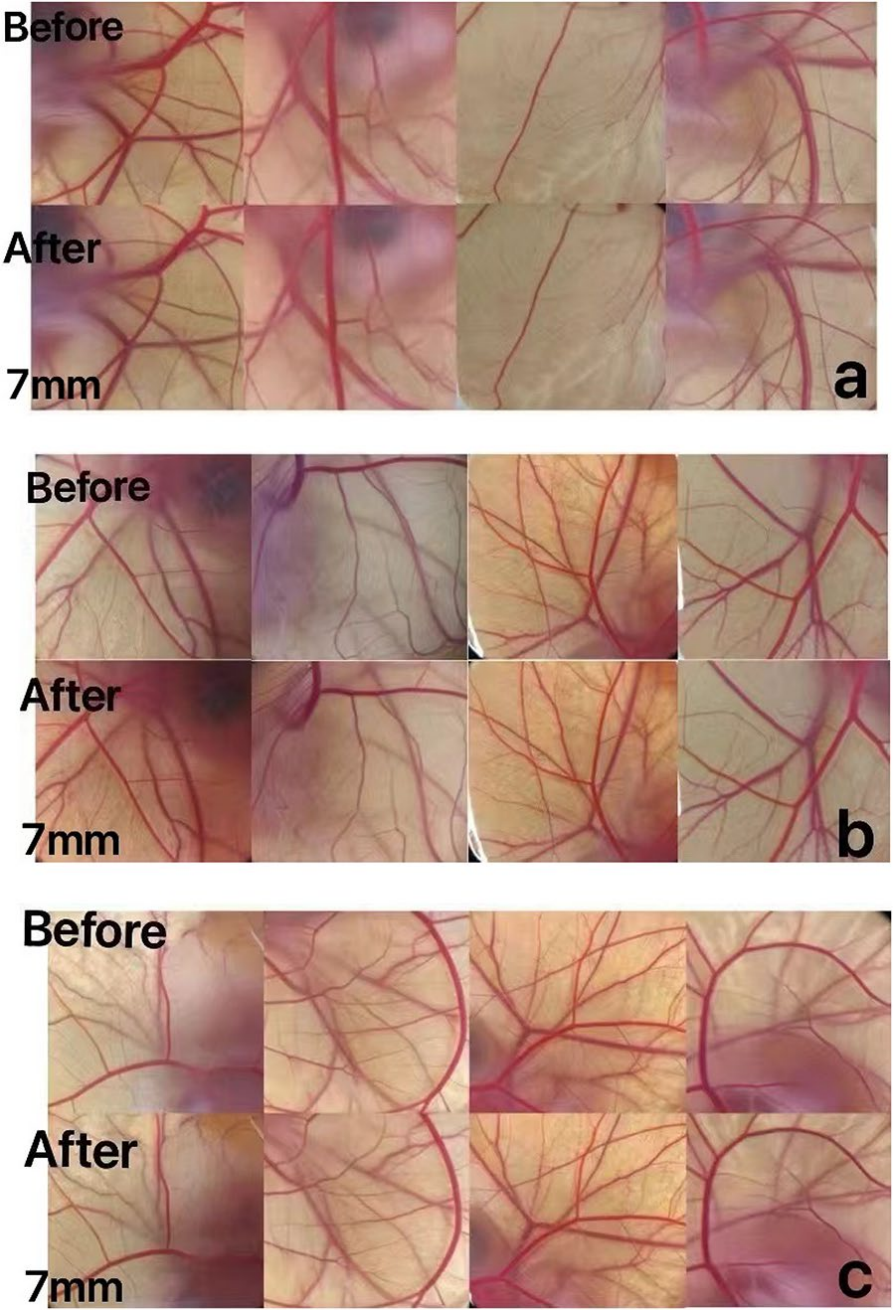
Comparison of samples before and after action. **a** Comparison before and after L0 action (local) **b** Comparison before and after L1 action (partial). **c** Comparison before and after L2 action (local). The distance between the objective lens and the object is 7 mm

HET-CAM is suitable for the evaluation of cosmetic products or raw materials (Vinardell et al. 1994). The experimental results showed in Fig. 11, three samples did not irritate the blood vessels, so the combination of the two experiments indicated the final conclusion that these samples did not have any irritation effects.

## Discussion

*Lactobacillus plantarum* is an anti-inflammatory and anticancer antioxidant, often used in fermented foods. Cosmetics with *Lactobacillus plantarum* can inhibit the degradation of collagen, achieve anti-aging effects, reduce the production of ROS (Schwarzenberger et al.^2012^)_._*S. cerevisiae* is used for brewing wine, making bread, and treating indigestion. The two probiotics were combined with litchi seeds respectively to stimulate the proliferation of active ingredients and improve the efficacy level. Fermentation of a single raw material can maximize its nutritional value. Literature has shown (Bhuyar et al. 2021) that compared with water extract, the contents of liquiritin, quercetin, epicatechin, cataphthol and anthocyanin in samples after fermentation, although the metabolites of different fungi are slightly different, will increase. Flavonoids can play an anti-inflammatory role by down-regulating the content of inflammatory factors. According to the results, the content of polysaccharide in the sample group was also significantly increased, and literature (Chen et al. 2024) showed that polysaccharide can play an antioxidant role by regulating the expression of downstream antioxidant enzymes through endogenous anti-oxidative stress Nrf2/ARE pathway, thereby reducing the generation of free radicals. Two cell models were set up in this experiment, one after UVA irradiation to determine the scavenging ability of L0, L1, L2 on free radicals, the expression of reactive oxygen species and collagen content; one after lipopolysaccharide for induction, to determine whether the lychee seed fermentation liquid has anti-inflammatory efficacy by determining the content of inflammatory factors and mRNA expression. A general conclusion can be drawn: Lychee seed ferment can effectively repair the damage caused by UVA. The specific conclusion is that the content of polysaccharides, Total polyphenol, proteins and flavonoids in lychee seed fermentation broth is obviously much higher than that of lychee seed water extract. The expression of active content has good whitening, antioxidant, anti-aging, anti-inflammatory effects (Pacularu-Burada et al. 2024) Fermentation can effectively enhance the active content of raw materials, enhance its antioxidant efficacy, anti-inflammatory efficacy and so on.

Indicators of antioxidant efficacy are mainly in the scavenging effect on DPPH radicals and hydroxyl radicals. DPPH (1,1-diphenyl-2-picrylhydrazyl radical) and hydroxyl radicals are one of the more oxidising free radicals in living organisms, and when they are over-expressed they will lead to problems such as ageing, dull complexion, sensitivity and skin cancer (Jena et al.^2024^).The level of free radical elimination by lychee seed fermentation broth is positively correlated with concentration, and that of the broth prepared by *S. cerevisiae* fermentation was better. When using MTT assay to detect cell survival and growth, this method is highly sensitive, convenient, and fast (Fatemi et al. 2024). When the lychee seed fermentation broth samples were not added, it was found that the solution was highly toxic to cells. When they were added, the cell survival rate became higher. When the cells were irradiated by UVA, the cell survival rate was 37.01%.When the samples were added, the cell survival rate was significantly improved, indicating that the lychee seed fermentation broths could reduce UVA-induced damage to cells. When the sample volume fraction was 5%, the cell survival rate was the highest. Therefore, samples with a 5% volume fraction were selected for the subsequent determination of antioxidant capacity in vitro. Elastin (ELN) and collagen type I (COL-1) are indicators of skin aging (Fang et al.^2023^), and malondialdehyde (MDA) and reactive oxygen species (ROS) are indicators for testing whether the organism is peroxidised. The data showed that lychee seed aqueous extract and fermentation solution could enhance ELN and COL-1 content while reducing MDA and ROS content, and the ability of fermentation solution was much higher than Lychee seed water extract.

The anti-inflammatory efficacy of the fermentation broth of lychee seeds is much higher than that of the water extract, mainly in the expression of inflammatory factors. Inflammatory factors act as triggers of the inflammatory response and participate in the body’s immune response. IL-6, COX-2, INOS, and TNF-a are all important inflammatory cytokines, and their excessive expression will cause the occurrence of inflammation in the body (Chang et al. 2023). IL-6 and TNF-a are pro-inflammatory factors involved in cell metabolism, and they work together to promote the occurrence and development of inflammatory response (León et al.^2023^) In normal cell tissues, COX-2 activity is extremely low, and when cells are stimulated by inflammation, COX-2 content will increase rapidly (Ahmadi et al. 2022) When inflammation occurs, iNOS will be induced to express and produce nitric oxide (NO), high concentration of NO will produce toxicity. The lychee seed water extract and fermentation broths could significantly reduce the content of inflammatory factors so as to achieve anti-inflammatory effects. Caspase-3, Caspase-9, and Bax are pro-apoptotic proteins, and studies (Cai et al.^2019^) have shown that they can directly or indirectly regulate cell apoptosis, while their excessive expression will lead to apoptosis. The experiments proved that compared with the model group, the sample group could significantly reduce the gene content of pro-apoptotic factors. Safety is very important for cosmetics. In this study, the red blood cell hemolysis test and chicken embryo allantoic membrane eye stimulation test were adopted to explore whether lychee seed fermentation broths were non-irritating and whether they could be applied to the human body. The results showed that there was no irritation in the samples.

## Conclusion

According to the above experimental data, it is clear that the lychee seed fermentation broths have great potential to be used in cosmetics as raw materials with anti-aging, anti-inflammatory, antioxidant, and other effects. This effect may be achieved by modulating and improving some indicators of aging and inflammation-related substances in the skin.

## Data Availability

The datasets used during the current study are available from the corresponding author on reasonable request.
